# Improved accuracy and reduced uncertainty in greenhouse gas inventories by refining the IPCC emission factor for direct N_2_O emissions from nitrogen inputs to managed soils

**DOI:** 10.1111/gcb.15884

**Published:** 2021-09-25

**Authors:** Kristell Hergoualc’h, Nathan Mueller, Martial Bernoux, Äsa Kasimir, Tony J. van der Weerden, Stephen M. Ogle

**Affiliations:** ^1^ Center for International Forestry Research (CIFOR) Lima Peru; ^2^ Department of Ecosystem Science and Sustainability Colorado State University Fort Collins Colorado USA; ^3^ Department of Soil and Crop Sciences Colorado State University Fort Collins Colorado USA; ^4^ Food and Agriculture Organization of the United Nations (FAO) Rome Italy; ^5^ University of Gothenburg Gothenburg Sweden; ^6^ Invermay Agricultural Centre AgResearch Ltd Mosgiel New Zealand; ^7^ Natural Resource Ecology Laboratory Colorado State University Fort Collins Colorado USA

**Keywords:** agriculture, anthropogenic emissions, climate change, fertilizer, greenhouse gas, manure, nitrous oxide, organic, soil, synthetic

## Abstract

Most national GHG inventories estimating direct N_2_O emissions from managed soils rely on a default Tier 1 emission factor (EF_1_) amounting to 1% of nitrogen inputs. Recent research has, however, demonstrated the potential for refining the EF_1_ considering variables that are readily available at national scales. Building on existing reviews, we produced a large dataset (*n* = 848) enriched in dry and low latitude tropical climate observations as compared to former global efforts and disaggregated the EF_1_ according to most meaningful controlling factors. Using spatially explicit N fertilizer and manure inputs, we also investigated the implications of using the EF_1_ developed as part of this research and adopted by the 2019 IPCC refinement report. Our results demonstrated that climate is a major driver of emission, with an EF_1_ three times higher in wet climates (0.014, 95% CI 0.011–0.017) than in dry climates (0.005, 95% CI 0.000–0.011). Likewise, the form of the fertilizer markedly modulated the EF_1_ in wet climates, where the EF_1_ for synthetic and mixed forms (0.016, 95% CI 0.013–0.019) was also almost three times larger than the EF_1_ for organic forms (0.006; 95% CI 0.001–0.011). Other factors such as land cover and soil texture, C content, and pH were also important regulators of the EF_1_. The uncertainty associated with the disaggregated EF_1_ was considerably reduced as compared to the range in the 2006 IPCC guidelines. Compared to estimates from the 2006 IPCC EF_1_, emissions based on the 2019 IPCC EF_1_ range from 15% to 46% lower in countries dominated by dry climates to 7%–37% higher in countries with wet climates and high synthetic N fertilizer consumption. The adoption of the 2019 IPCC EF_1_ will allow parties to improve the accuracy of emissions’ inventories and to better target areas for implementing mitigation strategies.

## Graphical abstract

The EF1 emission factor for direct N_2_O emissions from nitrogen inputs to managed soils as influenced by climate, management practices (fertilizer form, N application rate), land cover, and topsoil properties (texture class, C content, alkalinity) (a). The EF1 in wet or dry climates as influenced by the same variables (b). Means and 95% confidence intervals. 2006 IPCC is the default value from the 2006 IPCC guidelines.

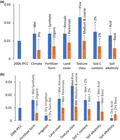

## 1 INTRODUCTION

Nitrous oxide (N_2_O) is a potent greenhouse gas (GHG) whose atmospheric concentration's rate of increase has more than quintupled from 0.15 ppbv year^−1^ a century ago to 0.85 ppbv year^−1^ in 2001–2015 (Wells et al., 2018). The primary source of this increase is the land and not the oceans, as suggested by changes in nitrogen (N) isotopic composition of atmospheric N_2_O (Jia et al., 2019). According to modeling estimates and global databases, agriculture is accountable for about two‐thirds of terrestrial emissions releasing over 6 Tg N_2_O year^−1^ in 2010–2016 (Jia et al., 2019). N_2_O emissions from the agricultural sector reported in national GHG communications include three main categories: manure management, managed soils, and biomass burning. Managed soils were estimated to contribute as much as 35%–86% to agricultural N_2_O emissions depending on the region (Janssens‐Maenhout et al., 2019). Emissions from managed soils occur directly as the result of N application and indirectly following leaching and runoff of applied N and deposition of volatilized anthropogenic N additions. As worldwide use of N fertilizer continues to increase (Janssens‐Maenhout et al., 2019) and fertilizer‐derived N_2_O emissions keep growing (Tian et al., 2020), estimating national N_2_O emissions from managed soils accurately is a cornerstone to improving global GHG emissions and testing the effectiveness of options for N_2_O emissions abatement.

The 2006 IPCC guidelines for national GHG inventories provide methodological guidance for estimating direct N_2_O emissions from contrasting soil, crop, or N source situations (eq. 11.1 in De Klein et al., 2006). The Tier 1 EF_1_ emission factor serves for quantifying direct N_2_O emissions resulting from fertilizer application, crop residues return to soils, and decomposition of soil organic matter (SOM) of mineral soils. Direct emissions from SOM decomposition of organic soils, application of N inputs on flooded rice fields, and deposition of urine and dung N on pasture, range, and paddock by grazing animals are estimated through other emission factors. The Tier 1 EF_1_ was set by De Klein et al. (2006) at 1% of the N either added and returned to soils or mineralized by soils with a confidence interval of [0.3%; 3%] according to findings by Bouwman and Boumans (2002), Bouwman et al. (2002b), Novoa and Tejeda (2006), and Stehfest and Bouwman (2006). The EF_1_ emission factor has been criticized for having been derived from a dataset biased toward mid‐latitude and temperate regions, being too uncertain, not accounting for differences in environmental conditions, management practices and land use systems, and assigning a linear response of N_2_O emissions to N inputs (Charles et al., 2017).

Emissions of N_2_O from soils result from complex interactions of production, consumption, and gas transport processes, which are controlled by biotic and abiotic factors (Butterbach‐Bahl et al., 2013). Nitrous oxide is predominantly formed and consumed by oxidation of ammonium (NH_4_^+^) through nitrification and reduction of N oxides (nitrate ${\text{NO}}_{3}^{‐}$, nitrite ${\text{NO}}_{2}^{‐}$) via denitrification (Hergoualc'h et al., 2007). Rates of nitrification and denitrification at the cellular level are governed primarily by the availability of N, oxygen, and organic carbon (C; Firestone & Davidson, 1989). These controls are affected by numerous properties of the ecosystem and their dynamics (e.g., edaphic properties, climate, plant–microbe interactions) which can exert synergistic or antagonistic influences on the emissions (Butterbach‐Bahl et al., 2013; Skiba & Smith, 2000). This complexity results in extreme spatiotemporal variability of N_2_O fluxes at the soil–atmosphere interface often leading to the presence of hot spots and occurrence of hot moments (Groffman et al., 2009; Hénault et al., 2012). Therefore, upscaling N_2_O emissions to national scales and developing emission factors for estimating national emissions with top‐down commodity data, such as national fertilizer consumption statistics, remain a challenge (Butterbach‐Bahl et al., 2013; Ogle et al., 2013).

The Tier 1 EF_1_ allows countries to compute direct N_2_O emissions from managed soils using national data on synthetic and organic N applied to soils, N in crop residues returned to soils, and N mineralized in inorganic soils. This emission factor has been historically derived from experiments looking at the response of N_2_O emissions to N fertilizer application as they outnumber studies examining N_2_O emissions from SOM mineralization or from crop residues returned to soils. While the N application rate is recognized as the best single predictor of N_2_O emissions induced by N fertilization (Albanito et al., 2017; Shcherbak et al., 2014), factors such as climate, edaphic properties, or management practices under various land use systems may interact to a great extent. For instance, Charles et al. (2017) found that the EF_1_ specific to organic N fertilizers increased by a factor of five as annual precipitation increased from below 250 mm to above 500 mm. The EF_1_ was also found to be influenced by soil properties including C content, texture, and pH, both globally and in national‐scale analyses (Charles et al., 2017; Rochette et al., 2018; Shcherbak et al., 2014). Crop type and fertilizer type modulated the EF_1_ computed from global data (Shcherbak et al., 2014), and data from the tropics (Albanito et al., 2017) and Mediterranean climates (Cayuela et al., 2017). Management practices including irrigation or the frequency of fertilizer application (Cayuela et al., 2017; Shcherbak et al., 2014) or parameters linked to the experimental design for measuring the fluxes such as the length of the experiment or the chamber size (Albanito et al., 2017; Shcherbak et al., 2014) were also found to influence the EF_1_. The literature, however, is divided on the type of response of the EF_1_ to the N application rate. A response faster than linear has been highlighted at a global scale on yearly fluxes following the application of synthetic fertilizers to various crop types (Gerber et al., 2016; Philibert et al., 2012; Shcherbak et al., 2014) and at local scales for specific crops in the period following N application (Hoben et al., 2011; Oktarita et al., 2017). In contrast, findings by other studies conducted at regional scales (Tropics, Mediterranean climate) or national scales do not support the hypothesis of a nonlinear increase in the annual EF_1_ as a function of the N applied (Albanito et al., 2017; Cayuela et al., 2017; Rochette et al., 2018).

The main objective of this research was to refine the IPCC Tier 1 EF_1_ emission factor for N_2_O emissions making use of the most recent scientific literature, and considering the influence of climate, management practices, land cover, and edaphic properties. Our approach consisted in compiling and combining existing datasets of EF_1_ and controlling variables, retaining only cases for which the EF_1_ was based on an unfertilized control site. We classified climate as wet or dry according to the definition adopted by the IPCC (Reddy et al., 2019). Management practices included N fertilizer type (organic, synthetic, mixtures of synthetic and organic forms), N application rate, and irrigation in dry climate. Land cover entailed annual croplands, bare soils, and perennial systems. Edaphic properties included variables related to texture (fine vs. medium and coarse), C content, and alkalinity. We also tested the potential of the experimental length of individual observations to modulate the EF_1_. A second objective of this research was to assess the implications of using the EF_1_ disaggregated by climate and fertilizer form from this research and adopted by the 2019 Refinement to the 2006 IPCC guidelines in place of the generic 1% value on direct soil N_2_O emissions from N inputs to global croplands.

## 2 MATERIALS AND METHODS

### 2.1 Selection of studies and extraction of data

We extracted all studies from the databases by Stehfest and Bouwman (2006; global dataset dominated by observations in Europe), van Lent et al. (2015; dataset for the tropics), Grace et al. (2016; dataset for Oceania), van der Weerden et al. (2016; dataset for Oceania), Albanito et al. (2017; dataset for the tropics), Cayuela et al. (2017; dataset for Mediterranean climate), Liu et al. (2017; global dataset), and Rochette et al. (2018; dataset for North America) to cover a broad range of environmental conditions and practices. We excluded studies which:

- Were from non‐peer‐reviewed publications,
- Were conducted in the laboratory or greenhouses, and modeling studies (only field studies were selected),
- Were conducted in flooded rice fields (emissions from N inputs in flooded rice are estimated using the IPCC EF_1FR_),
- Related to grazed soils where urine and/or dung was deposited (emissions from urine/dung inputs in grazed soils are estimated using the IPCC EF_3PRP_),
- Related to enhanced efficiency synthetic or organic fertilizer either treated with inhibitors or coated, and
- Were conducted on drained and/or managed organic soils (the EF_1_ serves for quantifying N_2_O emissions from SOM decomposition in mineral soils).

We further selected the cases from the source databases for which an emission factor was measured or could be computed from a control plot as:

$${\text{EF}}_{1i}=\frac{N_{2}O_{\text{Ti}}‐N_{2}O_{\text{Ci}}}{N_{i}},$$

where N_2_O_Ti_ is the N_2_O flux during the experimental period due to the application of inputs N_i_ and other unquantified sources of N, and N_2_O_Ci_ is the N_2_O flux during the experimental period at a control plot due to other sources of N than N_i_.

**FIGURE 1:**
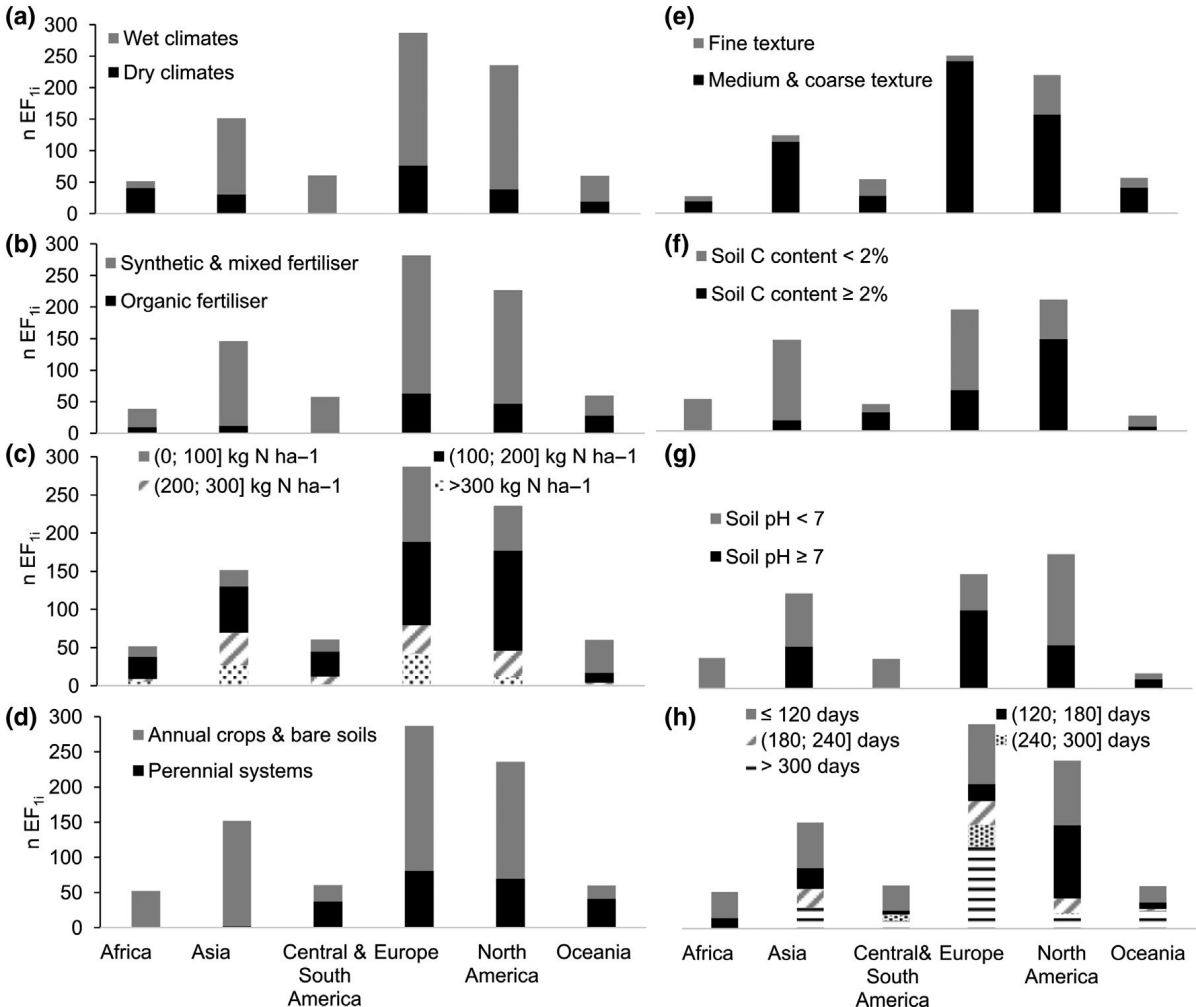
Frequency of the EF_1_ in the dataset among geographical regions according to climate (a), N fertilizer form (b), N application rate (c), land cover (d), soil texture (e), soil C content (f), soil pH (g), and length of the experiment (h)

The resulting database comprised 848 EF_1_ observations distributed globally (Figure 1a; Section 3.1).

### 2.2 Classification of variables influencing the emission factor

Among the variables that were present in the final database and deemed important controlling factors of the EF_1_, we selected those considered the most readily available to countries for conducting national inventories. These factors were related to climate, management practices, land cover, and edaphic properties in the topsoil, and were grouped into classes based on the following criteria.

**FIGURE 2:**
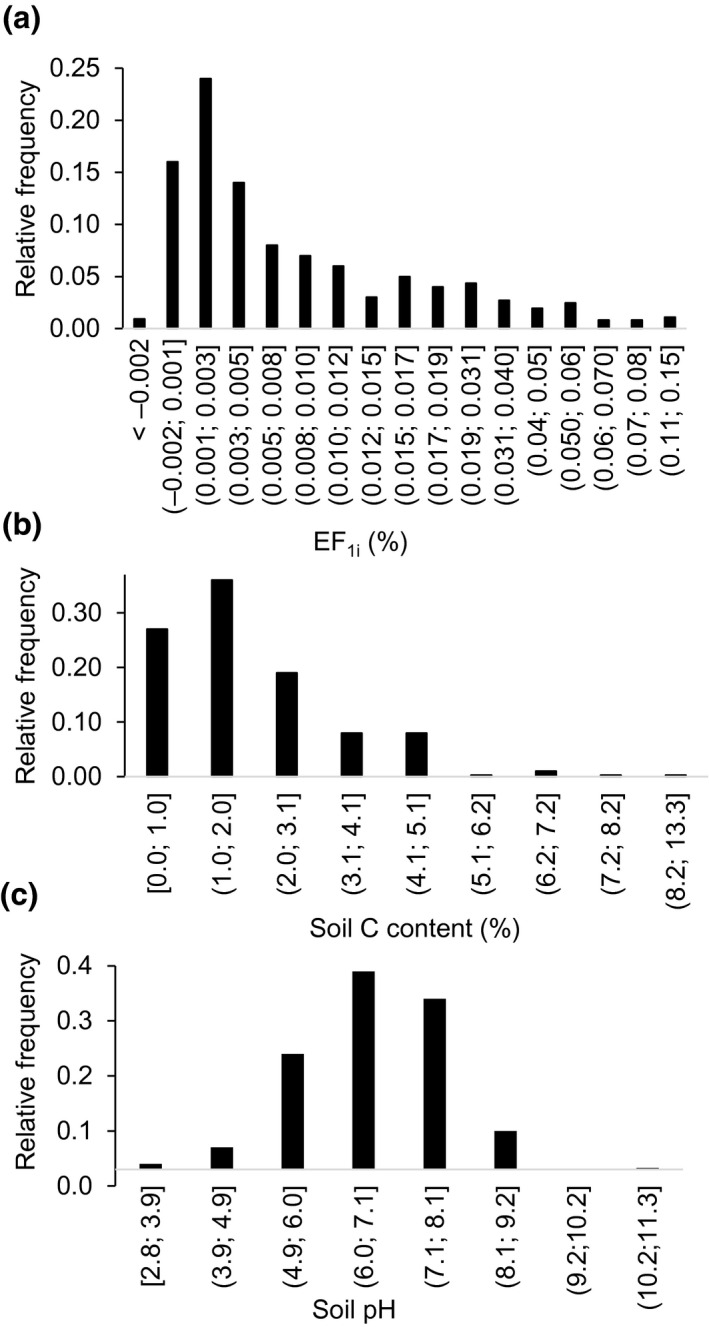
Relative frequency of the EF_1i_ emission factor (a), soil C content (b), and pH (c) in the dataset

- Climatic region: Wet or dry. Climate classification initially comprised four classes: temperate/boreal wet, temperate/boreal dry, tropical wet, and tropical dry. It was simplified by distinguishing dry climates from wet climates regardless of latitude since the EF_1_ in temperate/boreal and tropical areas either wet or dry were not significantly different from each other (Table S1). Temperate, boreal, and tropical zones correspond to those defined in chapter 3 of volume 4 in the 2019 IPCC refinement report (Reddy et al., 2019). Wet climates occur in temperate and boreal zones where the ratio of annual precipitation: potential evapotranspiration >1, and tropical zones where annual precipitation >1000 mm. Dry climates occur in temperate and boreal zones where the ratio of annual precipitation: potential evapotranspiration <1, and tropical zones where annual precipitation <1000 mm. Climate was assigned based on the coordinates provided in the studies.
- N fertilizer type: Synthetic fertilizer and mixtures of synthetic and organic forms (further referred to as synthetic and mixed fertilizer) or organic fertilizer. The influence of the fertilizer type was first tested using three classes: synthetic, organic, and mixtures of synthetic and organic forms. As the classes synthetic fertilizer and mixtures of synthetic and organic forms yielded similar EF_1_ values (Table S1), they were merged into a single class.
- N application rate: (0; 100], (100; 200], (200; 300] and >300 kg N ha^−1^ period^−1^. Intervals were built from the data distribution following the classification by Albanito et al. (2017).
- Water management: Irrigation or the absence of irrigation in dry climate.
- Land cover: Annual croplands and bare soils or perennial systems. Bare soils included 70% of bare soils and 30% of crops classified as undefined in the original databases. Perennial systems encompassed perennial croplands, grasslands, agroforestry systems, tree plantations, and managed forests. A preliminary analysis demonstrated a similar response of the EF_1_ for the classes of annual croplands, bare soils, and perennial systems (Table S1). Because vegetation cover over time for annual croplands and bare soils are closer to each other than long‐term vegetation cover in perennial systems, the first two classes were grouped into a single class.
- Soil texture class: Fine or medium coarse. Following the USDA classification system (USDA, 2017), fine‐textured soils included sandy clay, silty clay, and clay; medium‐textured soils were sandy loam, loam, silt loam, silt, clay loam, sandy clay loam, and silty clay loam; coarse‐textured soils comprised sand and loamy sand. The EF_1_ for medium‐ and coarse‐textured soils were similar (Table S1); therefore, these classes were grouped together.
- Soil C content: Low–medium (<2%) or high (≥2%). The initial analysis showed uniformity in mean EF_1_ for low (<1%) and medium (1%–2%) soil C contents (Table S1) suggesting disaggregating in two classes. The intervals were selected according to data distribution (Figure 2b) and following the classification by Cayuela et al. (2017).
- Soil alkalinity: acid (pH < 7) and basic (pH ≥ 7), as per data distribution (Figure 2c) and the classification by Shcherbak et al. (2014).

Several key controlling factors available at (sub)national level which were part of the original databases are not presented because either they had no significant influence on the EF_1_ (e.g., soil C:N ratio) or they were seldom reported (e.g., cation exchange capacity).

Some studies noted an influence of sampling‐related factors on the EF_1_. In particular, Albanito et al. (2017) found that the EF_1_ decreased below 1% in studies longer than 6 months. Therefore, we tested the potential effect of the experimental length of individual experiments on the EF_1_. We considered the length intervals ≤120, (120; 180], (180; 240], (240; 300], and >300 days, according to data distribution (Figure 1h) and following the classification by Albanito et al. (2017). Other sampling‐related factors like chamber size or time elapsed since last N application could not be tested given the scarcity in reporting these variables in original databases.

### 2.3 EF_1_ data analysis

We used linear mixed‐effect modeling (Gałecki & Burzykowski, 2013) for testing the response of the EF_1_ emission factor to climate, management practices, land cover, edaphic properties, and experimental length. This approach was selected to account for lack of independence among data from individual sites compared to data from different sites. A location identification was assigned to all individual observations from experimental sites. Observations either with an identical coordinate or being from the same bibliographic reference with a same soil type and a same land cover were considered a unique location for the analysis.

The models included location identification as a random effect, and climate, management practice, land cover, edaphic property, or experimental length as fixed effects. Means for the fixed effects were compared using the LSD Fisher test. The 95% confidence interval of fitted values by the models was considered for uncertainty quantification of the EF_1_. For each model, we report the level of significance, the root mean square error (*R* ^2^), which indicates the coincidence between observed and simulated EF_1_ values and the Akaike information criterion (AIC) for performance evaluation, where a smaller AIC is better. The statistical analysis was performed using the software Infostat (Di Rienzo et al., 2017).

The influence of controlling factors on the EF_1_ was first evaluated independently for each variable. Thereafter, considering that climate is the most readily available information to countries, the influence of each individual factor was tested by climate. To maximize the statistical power and minimize the bias in the estimates and errors of the fixed effects, we limited the analysis to sample sizes >20 (Bell et al., 2010; Hox, 1998).

### 2.4 Testing the implications of using the EF_1_ disaggregated by climate and fertilizer form in place of the 1% EF_1_ on direct soil N_2_O emissions from global agricultural croplands

The 2019 Refinement to the 2006 IPCC guidelines on National GHG Inventories offers countries the possibility to report their direct soil N_2_O emissions from N fertilizer application disaggregating them by climate and fertilizer form (table 11.1 in chapter 11 by Hergoualc'h et al., 2019). To understand the implications of substituting the EF_1_ from the 2019 IPCC Methods Refinement (further referred to as 2019 IPCC MR) for the 1% EF_1_ from the 2006 IPCC guidelines (further referred to as 2006 IPCC GL), we applied them to synthetic N fertilizer application rates and consumption data by Mueller et al. (2012) and manure application rates by West et al. (2014) from circa 2000, and computed direct soil N_2_O emissions from global agricultural croplands. Flooded rice was discarded from these datasets using the MIRCA2000 irrigation data (Portmann et al., 2010), since emissions from this crop are not assessed using the EF_1_. The Mueller et al.’s dataset of synthetic N application is spatially disaggregated and fused national and, where available, subnational data (see Table S2 in the paper by Mueller et al., 2012). The West et al. (2014) manure dataset elaborated on the gridded world livestock density distributed proportionally to the mix of cropland and pasture. The combined dataset comprises N application rates for 172 crops in 188 countries. Direct soil N_2_O emissions were estimated using a Monte Carlo analysis based on total N consumption (synthetic and manure) by grid cell and triangular probability distribution functions for the EF_1_ from our analysis. The variation in climate across individual countries (wet vs. dry) was based on the classification provided in the 2019 IPCC MR (Reddy et al., 2019). The uncertainty in emission was estimated as 95% confidence intervals by selecting the 2.5 and 97.5 quantiles in the distributions. This analysis was conducted in R (R Core Team, 2020).

**FIGURE 3:**
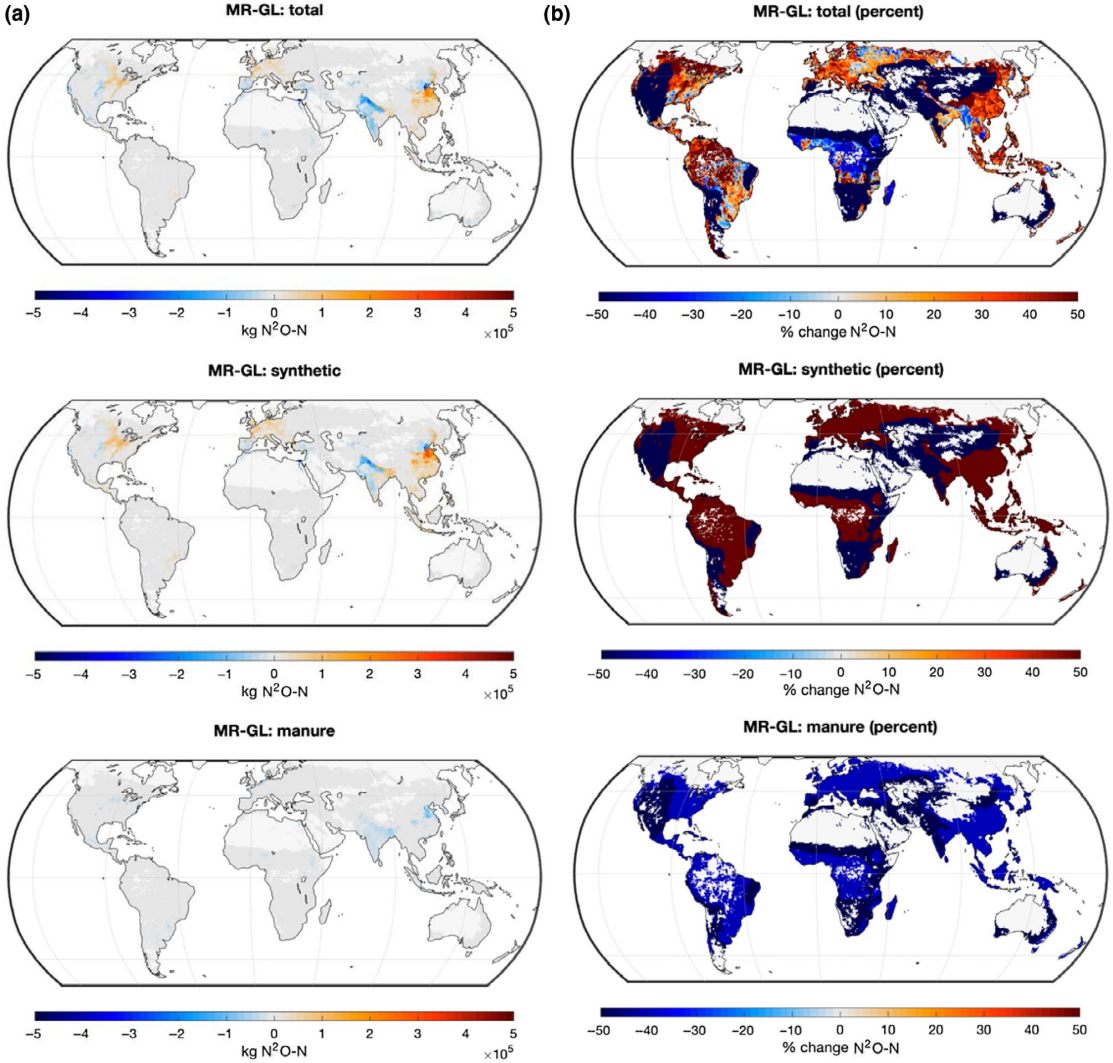
Absolute difference (a) and percentage difference (b) between direct soil N_2_O emissions from global agricultural croplands using the Tier 1 method from the 2019 IPCC Methods Refinement to the 2006 IPCC National GHG Inventories Guidelines (MR; Figure S1a) and the 2006 IPCC National GHG Inventories Guidelines (GL; Figure S1b). The top figures display emissions difference from both synthetic and manure application (total), the middle and bottom figures refer to synthetic and manure application separately

We produced maps of direct soil N_2_O emissions from global agricultural croplands using the Tier 1 method from the 2019 IPCC MR and the 2006 IPCC GL (Figure S1) and their absolute and percentage difference (Figure 3). These maps are presented for total (synthetic and manure) N application and for synthetic and manure application separately. Tabulated results present direct soil N_2_O emissions from global agricultural fertilizer N consumption and for the top 10 countries with the largest inputs of fertilizer N to croplands (Table S2).

## 3 RESULTS

### 3.1 Description and representativeness of the EF_1_ dataset

The EF_1i_ (*n* = 848) were in the range [−0.016; 0.147] and were 70% below 0.01 (Figure 2a). The dataset was unbalanced in geographical coverage and representation of controlling variables. It was dominated by cases from Europe (34%) and North America (28%), followed by Asia (18%) while Africa, Central‐South America, and Oceania formed an equal share of the dataset (6%−7%; Figure 1a). Most studies (76%) were conducted in wet climates except for Africa where the trend was opposite.

Organic and synthetic fertilizers varied by form and rate. The share of research in the dataset evaluating the response of the EF_1_ to organic fertilizer application was limited, except for Oceania (Figure 1b). Organic fertilizers were 33% animal slurry, 31% solid manure, 15% wastewater, and the remaining included liquid manure, compost, crop residues, and other forms. Among the treatments in our dataset, 56% of them applied organic fertilizer in a liquid form and qualified as high risk by Charles et al. (2017), 40% applied organic fertilizer in a solid form (medium–low risk), and 4% were unspecified. Synthetic fertilizers were 25% urea, 23% ammonium nitrate, 20% mixes, and the remaining encompassed anhydrous ammonia and other common mixes such as urea–ammonium–nitrate or calcium–ammonium–nitrate. In addition, 74% of N application rates in the dataset were below 200 kg N ha^­1^; however, Asia (especially China) displayed a greater proportion of studies with high N application rates (46% >200 kg N ha^­1^) in comparison with other regions (Figure 1c).

Perennial systems were not well represented (Figure 1d) and mostly comprised grasslands for harvesting (88%) and tree plantations (12%, e.g. pine plantations). Annual crops were dominated by wheat (24%) and maize (23%), followed by barley and maize (10% each). The EF_1i_ were essentially from medium‐ and coarse‐textured soils, though in Central and South America, texture was evenly distributed among classes (Figure 1e). Soil C contents varied from 0.03% to 13.3% with 63% below 2%, and all soils with C content >8% were Andosols (Figure 2b). Observations from low C content soils were more common apart for North America (Figure 1f). The dataset included more measurements on acid soils than on basic soils except for Europe and Oceania (Figure 1g). Soil pH values ranged from 3.2 to 11.3, with 67% in the range [6; 8] (Figure 2c). In terms of experimental design, 61% of studies were conducted over a period shorter than 180 days; longer studies were more frequent in Europe than elsewhere (Figure 1h).

### 3.2 Controlling factors of the EF_1_

Climate was a key control of the EF_1_ with a mean three times higher in wet climates than in dry climates (Table 1). In terms of management practices, the EF_1_ for synthetic and mixed fertilizers was double that of the EF_1_ for organic fertilizers while the rate of N application had no effect on the emission factor (*p* = .0639). The land cover also influenced the EF_1_ with a larger mean for annual croplands and bare soils than for perennial systems, but the level of significance of the model (*p* = .0235) was not as high as for the climate and fertilizer form models (<.0091). Edaphic properties modulated the EF_1_ with values two times higher in fine‐textured soils than in medium‐ and coarse‐textured soils, in C‐rich soils than in soils with low to medium C content, and in acid soils than in basic soils. The models for texture and soil C were highly significant (<0.0001) with an AIC below 3000. Finally, the analysis indicated a significant but unspecific response of the EF_1_ to the experimental length, with shortest (≤120 days) and longest (>300 days) experiments displaying a similar EF_1_ (0.012–0.013) and no tendency toward lower EF_1_ with increasing experimental length or vice versa. Each of the previously described models explained reasonably well the variation of the EF_1_ (.4 ≤ *R* ^2^ ≤ .51).

**TABLE 1:** Sample size, mean, and uncertainty range of the EF_1_ as influenced by climate, management practices (fertilizer form, N application rate), land cover, topsoil properties (texture class, C content, alkalinity), and experimental design (length of the experiment)

Factor	Class	*n*	Mean	95% CI	*p*	*R* ^2^	AIC
Climate	Wet	641	0.014^B^	0.011–0.017	.0090	.47	3384
	Dry	207	0.005^A^	0.000–0.011			
Fertilizer form	Synthetic and mixed	650	0.014^B^	0.0011−0.017	.0005	.49	3262
	Organic	162	0.007^A^	0.003−0.011			
N application rate	(0; 100] kg N ha^−1^	252	0.015^A^	0.011−0.018	.**0639**	.48	3391
	(100; 200] kg N ha^−1^	376	0.011^A^	0.007−0.014			
	(200; 300] kg N ha^−1^	131	0.013^A^	0.009−0.018			
	>300 kg N ha^−1^	89	0.010^A^	0.005−0.015			
Land cover	Annual croplands and bare soils	617	0.014^B^	0.011−0.017	.0235	.49	3387
	Perennial systems	231	0.009^A^	0.005−0.013			
Texture class	Fine	131	0.023^B^	0.018−0.028	<.0001	.49	2943
	Medium and coarse	601	0.010^A^	0.006−0.013			
Soil C content	High (≥2%)	265	0.015^B^	0.012−0.019	<.0001	.40	2491
	Low and medium (<2%)	400	0.007^A^	0.004−0.010			
Soil alkalinity	Acid soils (pH < 7)	392	0.013^B^	0.010−0.017	.0042	.40	2570
	Basic soils (pH ≥ 7)	273	0.006^A^	0.002−0.010			
Length of experiment	≤120 days	335	0.012^B^	0.008−0.015	<.0001	.51	3356
	(120; 180] days	183	0.02°C	0.016−0.024			
	(180; 240] days	84	0.009^B^	0.003−0.014			
	(240; 300] days	40	−0.002^A^	−0.010−0.007			
	>300 days	203	0.013^B^	0.009−0.017			

Note

A, B, C indicate a significant difference between means for a given factor based on LSD Fisher test. *p*, *R* ^2^, and Akaike information criterion (AIC) values indicate, respectively, the level of significance of the model, the coincidence between observed and simulated EF_1_ values, and the performance of the model (a smaller AIC is better). The *p* value of nonsignificant models is highlighted in bold.

Considering climate is the most accessible information to countries for conducting national GHG inventories, the influence of management practices, land cover, edaphic properties, and experimental design on the EF_1_ was tested by climate (Table 2). For several factors (N application rate, land cover, soil C content, experimental length), the sample size was too small for the analysis of dry climates therefore for these variables, the analysis was restricted to wet climates. The form of the fertilizer substantially influenced the EF_1_ in wet climates; with a similar response as when climates were aggregated, that is, a higher EF_1_ for synthetic and mixed fertilizers than for organic fertilizers. The N application rate did not affect the EF_1_ in wet climates, as indicated by the similarity in EF_1_ means. In dry climates, irrigation induced a higher EF_1_ than for rain‐fed lands. This dry climate EF_1_ in irrigated fields is very close to the dry climate EF_1_ regardless of irrigation (Table 1) as most dry climate observations were from irrigated lands (63%). The larger EF_1_ in fine‐textured soils than in medium‐ and coarse‐textured soils observed for all climates was persistent in wet climates whereas in dry climates, texture class did not significantly influence the EF_1_ (*p* = .1876). Similarly, the higher EF_1_ in C‐rich soils than in lower C soils was also significant when the data were limited to wet climates. Soil alkalinity modulated the EF_1_ in wet climates with higher values for acid soils, similarly as when climates were grouped together (Table 1). Interestingly, the pattern was opposite in dry climates, with a lower EF_1_ in acid soils than in basic soils. Lastly, the experimental length displayed no clear pattern on the EF_1_ in wet climates, similarly as for all climates (Table 1). In wet climates, the most significant models with highest *R* ^2^ were the ones using texture class (*p* < .0001, *R* ^2^ = .49) or fertilizer form (*p* = .0002, *R* ^2^ = .48) as a fixed effect; the one with the lowest AIC (1909) was the soil C content model, but it explained less variation in the EF_1_ (40%) than the aforementioned models. In dry climates, the model including irrigation was the most performant (AIC = 240) but displayed a relatively low *R* ^2^ (.30).

**TABLE 2:** Sample size, mean, and uncertainty range of the EF_1_ in wet or dry climates as influenced by management practices (fertilizer form, N application rate, irrigation), land cover, topsoil properties (texture class, C content, and alkalinity), and experimental design (length of the experiment)

Factor	Class	*n*	Mean	95% CI	*p*	*R* ^2^	AIC
Fertilizer form	Wet climate synthetic and mixed fertilizer	503	0.016^B^	0.013−0.019	.0002	.48	2601
	Wet climate organic fertilizer	109	0.006^A^	0.001−0.011			
	Dry climate synthetic and mixed fertilizer	147	0.005^A^	0.003−0.008	.**6544**	.37	467
	Dry climate organic fertilizer	53	0.005^A^	0.002−0.008			
N application rate^a^	Wet climate (0; 100] kg N ha^−1^	204	0.018^A^	0.013−0.022	.033	.47	2712
	Wet climate (100; 200] kg N ha^−1^	265	0.012^A^	0.007−0.016			
	Wet climate (200; 300] kg N ha^−1^	102	0.015^A^	0.010−0.020			
	Wet climate >300 kg N ha^−1^	70	0.011^A^	0.005−0.017			
Irrigation	Dry climate with irrigation	94	0.004^B^	0.003−0.006	.0088	.30	240
	Dry climate rain‐fed	56	0.001^A^	−0.001−0.003			
Land cover^a^	Wet climate annual croplands and bare soils	425	0.017^B^	0.013−0.021	.0049	.48	2707
	Wet climate perennial systems	216	0.010^A^	0.006−0.015			
Texture class	Wet climate fine texture	107	0.027^B^	0.021−0.033	<.0001	.49	2396
	Wet climate medium and coarse texture	461	0.011^A^	0.007−0.015			
	Dry climate fine texture	24	0.001^A^	−0.004−0.006	.**1187**	.29	383
	Dry climate medium and coarse texture	140	0.006^A^	0.003−0.008			
Soil C content^a^	Wet climate high soil C (≥2%)	256	0.016^B^	0.012−0.020	.003	.40	1909
	Wet climate low and medium soil C (<2%)	218	0.009^A^	0.005−0.013			
Soil alkalinity	Wet climate acid soils (pH < 7)	350	0.015^B^	0.011−0.019	.0165	.37	1962
	Wet climate basic soils (pH ≥ 7)	123	0.007^A^	0.002−0.013			
	Dry climate acid soils (pH < 7)	42	0.002^A^	−0.001−0.004	.0369	.20	418
	Dry climate basic soils (pH ≥ 7)	150	0.005^B^	0.003−0.007			
Length of experiment^a^	Wet climate ≤120 days	274	0.014^B^	0.010−0.018	<.0001	.50	2677
	Wet climate (120; 180] days	140	0.024^C^	0.019−0.030			
	Wet climate (180; 240] days	43	0.006^AB^	−0.002−0.014			
	Wet climate (240; 300] days	23	−0.001^A^	−0.011−0.009			
	Wet climate >300 days	158	0.015^B^	0.010−0.020			

Note

A and B indicate a significant difference between means for a given factor based on LSD Fisher test. *p*, *R* ^2^, and Akaike information criterion (AIC) values indicate, respectively, the level of significance of the model, the coincidence between observed and simulated EF_1_ values, and the performance of the model (a smaller AIC is better). The *p* value of nonsignificant models is highlighted in bold.

^a^ Sample sizes too small for dry climate.

Considering data availability at national level and the performance of the models, the dry climate EF_1_ (0.005, 95% CI 0.000–0.011, Table 1) and wet climate EF_1_ for synthetic and mixed fertilizer (0.016, 95% CI 0.013–0.019) and for organic fertilizer (0.006, 95% CI 0.001–0.011; Table 2) were deemed relevant for national GHG inventories by the 2019 Refinement to the 2006 IPCC guidelines. These emission factors have a much narrower uncertainty compared with the 2006 IPCC GL EF_1_ (0.01, 95% CI 0.003–0.03).

### 3.3 Implications of using the 2019 IPCC EF_1_ disaggregated by climate and fertilizer form in place of the 2006 IPCC 1% EF_1_ on direct soil N_2_O emissions from global agricultural croplands

Direct soil N_2_O emissions from global agricultural croplands estimated from the 2019 IPCC MR were 4% higher than emissions computed from the 2006 IPCC GL (Table 3, 1073 and 1030 Gg N_2_O‐N, respectively). Among the top three emitters—China, the United States, and India which all together contribute half of global emissions (Table 3), China and the United States emissions were, respectively, 21% and 13% higher when estimated from the 2019 IPCC MR than from the 2006 IPCC GL, a trend particularly pronounced toward the eastern wet areas of those countries (Figure 3a top panel). In India, which is predominantly dry, the 2019 IPCC MR emissions were 21% lower than the 2006 IPCC GL estimates. Importantly, the 2019 IPCC MR considerably reduced the uncertainty range of global emissions (883–1285 Gg N_2_O‐N) relative to the range computed from the 2006 IPCC GL (539–2713 Gg N_2_O‐N).

**TABLE 3:** Estimates and 95% confidence intervals for direct soil N_2_O emissions from global agricultural croplands in circa 2000, and also countries with the largest inputs of fertilizer N to croplands (synthetic and manure N)

	Direct soil N_2_O emissions (Gg N_2_O‐N)					
	Total fertilizer		Synthetic fertilizer		Manure fertilizer	
	Estimate	95% CI	Estimate	95% CI	Estimate	95% CI
Global agriculture						
2019 IPCC MR	1,073.3	883.2–1,284.9	882.0	740.8–1,036.6	191.3	92.3–296.0
2006 IPCC GL	1,030.1	539.1–2,712.7	696.2	364.4–1,833.5	333.9	174.7–879.2
China						
2019 IPCC MR	316.2	269.9–365.5	279.5	239.3–321.5	36.7	15.8–58.2
2006 IPCC GL	261.8	137.0–689.5	199.3	104–3–524.8	62.6	32.7–164.7
United States						
2019 IPCC MR	149.3	125.9–174.9	127.9	108.7–148.2	21.3	9.8–33.4
2006 IPCC GL	132.0	69.1–347.6	95.2	49.8–250.7	36.8	19.2–96.8
India						
2019 IPCC MR	118.6	82.8–161.7	86.9	63.4–114.9	31.7	15.9–49.9
2006 IPCC GL	150.6	78.8–396.6	93.1	48.7–245.2	57.5	30.1–151.3
Brazil						
2019 IPCC MR	31.5	29.7–51.5	23.1	19.8–26.4	8.4	3.6–13.3
2006 IPCC GL	29.5	15.4–77.6	15.2	7.9–40.0	14.3	7.5–37.7
Indonesia						
2019 IPCC MR	30.5	25.8–35.2	26.4	22.6–30.2	4.1	1.5–6.7
2006 IPCC GL	23.3	12.2–61.4	16.5	8.6–43.4	6.8	3.6–18.0
France						
2019 IPCC MR	30.3	25.9–34.8	27.3	23.4–31.3	3.0	1.1–4.9
2006 IPCC GL	22.1	11.5–58.1	17.1	8.9–45.0	5.0	2.6–13.1
Germany						
2019 IPCC MR	24.9	21.2–28.7	22	18.8–25.1	3.0	1.1–4.9
2006 IPCC GL	18.6	9.8–49.1	13.7	7.2–36.1	4.9	2.6–13.0
Canada						
2019 IPCC MR	23.4	19.9–27.1	21.2	18.1–24.5	2.2	1.0–3.4
2006 IPCC GL	19.4	10.1–51.0	15.6	8.2–41.2	3.7	2.0–9.8
Mexico						
2019 IPCC MR	17.6	13.1–22.8	12.5	9.9–15.5	5.1	2.6–7.9
2006 IPCC GL	20.8	10.8–55.3	11.7	6.1–31.0	9.1	4.7–24.3
Pakistan						
2019 IPCC MR	14.9	4.8–27.1	11.4	3.7–20.7	3.5	1.1–6.4
2006 IPCC GL	27.5	14.4–72.5	20.7	10.8–54.4	6.9	3.6–18.1

Note

Estimates are provided using the Tier 1 method from the 2019 IPCC Methods Refinement to the 2006 IPCC National GHG Inventories Guidelines (2019 IPCC MR) and the 2006 IPCC National GHG Inventories Guidelines (2006 IPCC GL).

Estimated global emissions from synthetic N fertilizer application increased by 27% with the use of the 2019 IPCC MR as compared to emissions computed from the 2006 IPCC GL (882 and 696 Gg N_2_O‐N, respectively). This tendency was not evenly distributed, with wet regions displaying a strong percentage increase and dry regions a strong percentage decrease (Figure 3b middle panel). Among the top 10 emitting countries, France, Indonesia, and Germany had the largest increase (+60%), and only Pakistan, which is dry, had a decrease in emissions (−45%). Emissions from China and the United States were increased by 40% and 34%; emissions from India were decreased by 7%.

Estimated global emissions from manure application to croplands were almost halved by using the 2019 IPCC MR instead of the 2006 IPCC GL (−43%), a trend consistent for all countries (Figure 3a bottom panel) and slightly more pronounced in dry regions than in wet regions (Figure 3b bottom panel). Countries with the largest decreased emissions included Pakistan (−49%) and India (−45%). The contribution of global manure‐derived emissions to global total emissions shifted from 18% to 32% when using the 2019 IPCC MR, as a result of a corresponding decreased share of emissions from synthetic fertilizer in dry areas.

## 4 DISCUSSION

### 4.1 Representativeness of the dataset, biases, and research directions

With 848 EF_1_ observations, the extended dataset covers a broad range of geographies, climates, management practices, land covers, and edaphic properties. It was produced by compiling existing databases, taking care to encompass regions like the tropics, Oceania, and dry climates formerly underrepresented in the Stehfest et al.’ database used to develop the 2006 IPCC GL EF_1_. However, even this updated large dataset remains unbalanced toward mid‐latitude northern temperate regions, reinforcing the need for additional research in some regions, especially in Africa and Central‐South America and in dry climates (Figure 2a).

While manure represents one‐third of total N application worldwide (Table S2), the EF_1i_ in the final dataset were essentially from experiments testing the response of N_2_O emission to synthetic and mixed N fertilizer (80%). Our dataset included most studies from the reviews by Charles et al. (2017) and Zhou et al. (2017) on organic amendments, and the inclusion of the few missing cases from these reviews would only enlarge marginally our dataset and reinforce its geographical unbalance. Thus, the lack of quantitative data on how organic fertilizers influence N_2_O emissions emerges as a research gap of global significance. Our dataset encompassed a wide range of N application rates ([13; 1670] kg N ha^−1^ period^−1^) and evaluating the representativeness of these rates is difficult as standard recommendations vary according to the land cover depending on the fertilizer form. Besides in places local practices adjust the rates to satisfy plant needs. Moreover, the experiments mostly focused on a single N application which amount does not necessarily reflect an annual application rate (e.g., for crops fertilized more than once a year), making a comparison with standard annual rates applied to croplands difficult.

Major global annual croplands like wheat, maize, and barley which all together account for 44% of global N inputs (West et al., 2014) were well represented in the dataset while there were relatively few soybean studies, which may not be surprising given the low amounts of fertilizer added to the N‐fixing soybeans. Perennials were underrepresented, especially key global crops like sugarcane and oil palm which expand rapidly over the tropics (Phalan et al., 2013; Skiba et al., 2020).

Dominant edaphic properties in the dataset (medium and coarse texture—Figure 1e, low‐medium C content—Figure 2b, and [6; 8] pH soils—Figure 2c) mirror characteristics of lands suitable and available for agriculture.

Some final conclusions drawn from the data compilation for this research point toward the necessity to maintain quality, credibility, and transparency standards in science. Several of the databases combined in the dataset were reduced, some of them to a great extent to meet the IPCC quality criteria of selecting peer‐reviewed works published in scientific journals. Also, some syntheses that did not fully disclose data sources were discarded.

### 4.2 The EF_1_, its controls, and its uncertainty

Variations in the EF_1_ as affected by the environmental controls are consistent with our process understanding of soil–atmosphere N_2_O exchange. Soil N_2_O fluxes are largely controlled by soil moisture which regulates soil aeration and oxygen supply to microorganisms (Butterbach‐Bahl et al., 2013). N‐oxides are emitted predominantly in the form of nitric oxide (NO) below a soil water‐filled pore space (WFPS) of around 50%, above which N_2_O dominates over NO and reduces into N_2_ at high WFPS (Davidson et al., 2000). The higher EF_1_ in wet climates than in dry climates (Table 1) and in irrigated lands than in rain‐fed lands of dry climates (Table 2) are consistent with corresponding average WFPS and mechanisms governing nitrification and denitrification. Even though studies seldom reported soil moisture, the average WFPS was significantly higher in wet climates (58%, *n* = 123) than in dry climates (50%, *n* = 42; *p* = .0128) and in irrigated lands (58%, *n* = 22) than in rain‐fed lands (40%, *n* = 14) of dry climates (*p* = .0129). Furthermore, a higher EF_1_ with increased precipitation is aligned with findings by Charles et al. (2017) and the EF_1_ for dry climates (0.005, 95% CI 0.000–0.011, Table 1) is similar to the EF_1_ for Mediterranean climate computed by Cayuela et al. (2017) (0.005, 95% CI 0.004–0.006).

Regarding management practices, our results suggest a higher EF_1_ for synthetic and mixed fertilizers (0.0014) than for organic fertilizers (0.007) (Table 1) in wet climates (EF_1 Wet Synth&mix_ = 0.016, EF_1 Wet Org_ = 0.006) but not in dry climates where both fertilizer forms yielded a similar EF_1_ (0.005; Table 2). The EF_1 Wet Org_ is extremely similar to the value obtained by Charles et al. (2017; 0.0057) with a global dataset dominated by wet climate observations and by Zhang et al. (2020; 0.0056) with a Chinese wet climate‐dominated dataset. In contrast, Zhou et al. (2017) found a much higher EF_1_ for manure (0.0187) based on a dataset also essentially from wet climates. The discrepancy in the result by Zhou et al. (2017) may lie in differences with the other datasets in the chemical composition and state (raw or composted) of the manure, its application mode (surface or subsurface), and edaphic properties which all have been observed to influence the EF_1_ for organic fertilizer in different ways. A lower EF_1_ for organic fertilizer than for synthetic and mixed fertilizers has been attributed to the supply of organic C enhancing both N immobilization (hereby reducing substrate supply for nitrification and denitrification) and denitrification reduction of N_2_O to N_2_ (Zhou et al., 2017). This explanation supports the similarity in the EF_1_ among fertilizer forms in dry climate where denitrification is limited, a result also found by Cayuela et al. (2017).

Contrary to some studies (e.g., Gerber et al., 2016; Philibert et al., 2012; Shcherbak et al., 2014), the EF_1_ was not influenced by the rate of N application. Testing this response requires an EF_1i_ dataset with at least three different levels of N input per site (Shcherbak et al., 2014), which was not part of the objectives of our research. Instead, we aimed at covering a large range of geographies, management practices, land covers, and edaphic properties to refine the EF_1_ for use with national fertilizer consumption statistics. Nonetheless, we recommend countries with detailed fertilizer input rates test for an exponential response of the EF_1_ to N inputs and develop their own emission factor response curve. Furthermore, countries with detailed data on N in plants may also test for a response to N surplus (i.e., N applied minus N uptaken by plants) which was found by several studies (Eagle et al., 2020; van Groenigen et al., 2010) to be a better predictor of soil N_2_O emissions than the rate of N application.

Our results suggest a higher EF_1_ for annual croplands and bare soils than for perennial systems overall (EF_1 Annual_ = 0.014 vs. EF_1 Perennial_ = 0.009, Table 1) and in wet climates (EF_1 Annual_ = 0.017 vs. EF_1 Perennial_ = 0.010, Table 2). This result is aligned with findings by Abalos et al. (2016) in Ontario, Canada, who found EF_1_ 3.7, 3.1, and 1.3 times higher for annual crops than for perennial crops in three consecutive years. The difference in structure and functioning between annual and perennial systems induces distinct soil moisture and nutrient availability patterns and also affects soil microbial community composition (Abalos et al., 2016; Thompson et al., 2016). The permanence of perennial crops roots and their extended architecture maintains stable soil moisture levels over time (Vico & Brunsell, 2018) and favors soil organic matter buildup, which improves soil structure and reduces anaerobic microsites (Abalos et al., 2016). The synergistic influence of these factors leads to overall lower soil N_2_O emissions in perennial than in annual croplands. This difference is reinforced by the continuous activity of perennial systems throughout the year which, compared to annual crops, reduces soil N availability for microbial conversion to N_2_O (Abalos et al., 2016; Gelfand et al., 2016). Finally, owing to some of the aforementioned mechanisms, distinct N‐cycling microbial communities evolve in annual and perennial systems. A detailed description of differences in ammonia oxidizers and denitrifiers composition between annual and perennial croplands is provided by Thompson et al. (2016). As noted by Abalos et al. (2016), the potential for perennial systems to lower N_2_O emissions deserves further research attention; a conclusion greatly reinforced by the disproportion of annual versus perennial cropland studies in our dataset (Figure 1d).

Edaphic properties influence microbial nitrification and denitrification activity in several ways. Soil texture, in combination with soil bulk density and moisture, influences oxygen diffusion through the soil matrix (Butterbach‐Bahl et al., 2013). Generally, poorly drained fine‐textured soils favor N_2_O emissions while well‐drained coarse‐textured soils favor NO emissions (Bouwman et al., 2002a). This observation supports the larger EF_1_ in fine‐textured soils than in medium‐ and coarse‐textured soils overall (Table 1) and in wet climates (Table 2), which is also consistent with findings by Charles et al. (2017) and Rochette et al. (2018) when organic fertilizer is applied. The texture effect on the EF_1_ was insignificant in dry climates and potentially overridden by the climate effect leading to a dominance of NO emissions over N_2_O emissions regardless of texture. Notwithstanding, this result is based on a limited number of studies and needs further research of fine‐textured soil in dry climates in order to be conclusive.

Soil C plays a major role in N_2_O emissions as it serves as an electron donor for denitrification (Knowles, 1982), affects the water holding capacity and therefore the availability of oxygen in soils (Zhu et al., 2020), and stimulates heterotrophic respiration providing suboxic conditions for dissimilatory nitrate reduction pathways (Morley & Baggs, 2010). While some of these effects counter each other, the EF_1_ has generally been found to increase as soil C content reaches higher levels (Charles et al., 2017; Rochette et al., 2018; Shcherbak et al., 2014), which is consistent with our findings.

The control that the pH exerts on soil N_2_O emissions is complex and dependent on nutrient status (Granli & Bøckman, 1996). Globally, the EF_1_ increases with decreasing pH (Shcherbak et al., 2014; Wang et al., 2018), possibly as a result of the inhibition of N_2_O reduction into N_2_ during denitrification (Hénault et al., 2019). Conversely, where nitrification is the main N_2_O production pathway, emissions tend to increase as the pH increases, at least in the pH range 6–8 (Granli & Bøckman, 1996). While denitrification is believed to be the main N_2_O‐forming process, in dry climates, nitrification is likely to be more dominant. Therefore, the opposite response of the EF_1_ to the pH in wet (EF_1 acid_ > EF_1 basic_) and dry (EF_1 acid_ < EF_1 basic_) climates (Table 2) is coherent with current mechanistic understanding of nitrification and denitrification.

In their review of studies in the tropics, Albanito et al. (2017) observed a decrease in the EF_1_ below 1% in studies longer than 6 months and recommended to further evaluate the effect of study length on the response of N_2_O. Like Shcherbak et al. (2014) or Wang et al. (2018), we did not find a specific response of the EF_1_ to the experimental length. However, the lack of significance in our study may lie in the share of synthetic and organic fertilizers in the datasets, since organic fertilizers can be expected to mineralize slowly and release N_2_O over longer periods than synthetic fertilizers.

Finally, the uncertainty of the EF_1_ (Tables 1 and 2) is reduced compared to the 95% CI in the 2006 IPCC guidelines (0.003–0.03); as also found by Philibert et al. (2012) or Shcherbak et al. (2014) for a range of linear and nonlinear models. The CI of the EF_1_ for dry climate (0.000–0.011) is more conservative than the value obtained by Cayuela et al. (2017) for Mediterranean climate (0.004–0.006). The CI for the EF_1_ for organic fertilizer in wet climate (0.001–0.011) is consistent with the result by Charles et al. (2017) for organic amendment (0.000–0.012).

### 4.3 Implications of using the 2019 IPCC EF_1_ in place of the 2006 IPCC EF_1_

Robust estimates of soil direct N_2_O emissions from agricultural soils are essential not only for global GHG emission assessments but also for evaluating progress in reducing emissions with mitigation programs (Ogle et al., 2020). The 2019 IPCC MR Tier 1 EF_1_ offers the opportunity to improve the accuracy of global and country‐scale accounting of direct N_2_O emissions from agricultural soils using N consumption data disaggregated by climate and fertilizer form. We compared the emissions from global and national agricultural croplands in circa 2000 using subnational N fertilizer data and the two sets of EF_1_ factors from the 2006 IPCC GL and 2019 IPCC MR. Gerber et al. (2016) conducted a similar study for contrasting the response of N_2_O emissions to the 2006 IPCC GL EF_1_ and to an exponential model. Direct soil N_2_O emissions from global agriculture using the 2006 IPCC GL (1.0 Tg, Table 3) were much larger in our study than the estimate by Gerber et al. (2016), excluding flooded rice (0.73 Tg, their Table S2). The difference lies in the manure dataset used by Gerber et al. (2016) from Herrero et al. (2013) in which application rates are four times lower (7.8 Tg N) than the estimate by West et al. (2014), which we used in our analysis (33.9 Tg N, Table S2). According to a study on global N budget by Zhang et al. (2021), the West et al.’s (2014) data are on the higher end for manure N applied to cropland in the United States but are similar to the 2000 FAO data by Tubiello et al. (2013). Furthermore, our estimates of direct soil N_2_O emissions from global agriculture (Table 3) are two times lower than the 2.0 Tg computed by Tian et al. (2020) for the year 2000. The later includes emissions from N applied to flooded rice, from crop residue inputs, and from the decomposition of drained organic soils which together may account for the difference in estimates (Gerber et al., 2016; Tubiello et al., 2013).

Our results show that the use of the 2019 IPCC MR in place of the 2006 IPCC GL marginally increases soil direct N_2_O emissions from global agriculture but significantly reduces the uncertainty in the global estimate (Table 3). Removing the four countries among the top 10 emitting countries which report their emissions to the UN Framework Convention on Climate Change (UNFCCC) with Tier 2 or 3 methods, instead of Tier 1 (China, India, the United States, and Canada), suggests that global estimates do not change much when using the 2019 IPCC MR and the 2006 IPCC GL (466 Gg N for both). The national GHG communication to the UNFCCC of China (China, 2018) and India (Ministry of Environment & Forests, 2012) mention the use of country‐specific EF_1_ though the lack of reporting of values or disaggregation type limits an evaluation of how their emission factor compares with the 2019 IPCC MR or the 2006 IPCC GL EF_1_. The United States employs a model‐based approach (Tier 3) with the DayCent Ecosystem Model (US‐EPA, 2021), though emissions for croplands not simulated by the model are assessed based on the 2006 IPCC Tier 1 EF_1_. Given the large N consumption by the country, the adoption of the 2019 IPCC Tier 1 may to some extent alter estimates of global emissions that are reported to the UNFCCC. Canada uses a country‐specific EF_1_ which takes into account moisture regimes and topographic conditions (Canada, 2020), an approach similar to the 2019 IPCC MR. In general, the application of the 2019 IPCC MR will increase emission estimates for those countries with a predominantly wet climate and a large share of synthetic to manure fertilizer consumption such as France, compared to countries with a climate predominantly dry and a small share of synthetic to manure consumption, such as Mexico, and countries with dry climates such as Pakistan (Table 3; Table S2). The application of these factors in countries with a dry climate should be straightforward, while countries with wet or mixed climates, such as Indonesia and Brazil, will need their N consumption data to be disaggregated by fertilizer form and location. Rather than a constraint, however, this is an opportunity for countries to produce more accurate emission data, and better target mitigation strategies.

## CONFLICT OF INTEREST

The authors declare no conflict of interest.

## Supporting information

Supplementary Material

## Data Availability

The data that support the findings of this study are available at https://doi.org/10.17528/CIFOR/DATA.00273.

## References

[gcb15884-bib-0001] Abalos, D. , Brown, S. E. , Vanderzaag, A. C. , Gordon, R. J. , Dunfield, K. E. , & Wagner‐Riddle, C. (2016). Micrometeorological measurements over 3 years reveal differences in N_2_O emissions between annual and perennial crops. Global Change Biology, 22, 1244–1255. 26491961 10.1111/gcb.13137

[gcb15884-bib-0002] Albanito, F. , Lebender, U. , Cornulier, T. , Sapkota, T. B. , Brentrup, F. , Stirling, C. , & Hillier, J. (2017). Direct nitrous oxide emissions from tropical and sub‐tropical agricultural systems—A review and modelling of emission factors. Scientific Reports, 7(1). 10.1038/srep44235 PMC5345046 28281637

[gcb15884-bib-0003] Bell, B. A. , Morgan, G. B. , Schoeneberger, J. A. , Loudermilk, B. L. , Kromrey, J. D. , & Ferron, J. M. (2010). Dancing the sample size limbo with mixed models: How low can you go. SAS Global Forum, 4, 11–14.

[gcb15884-bib-0004] Bouwman, A. , & Boumans, L. (2002). Emissions of N_2_O and NO from fertilized fields: Summary of available measurement data. Global Biochemical Cycles, 16, 1058. 10.1029/2001GB001811

[gcb15884-bib-0005] Bouwman, A. F. , Boumans, L. J. M. , & Batjes, N. H. (2002a). Emissions of N_2_O and NO from fertilized fields: Summary of available measurement data. Global Biogeochemical Cycles, 16, 6‐1–6‐13.

[gcb15884-bib-0006] Bouwman, A. F. , Boumans, L. J. M. , & Batjes, N. H. (2002b). Modeling global annual N_2_O and NO emissions from fertilized fields. Global Biochemical Cycles, 16. 10.1029/2001GB001812

[gcb15884-bib-0007] Butterbach‐Bahl, K. , Baggs, E. M. , Dannenmann, M. , Kiese, R. , & Zechmeister‐Boltenstern, S. (2013). Nitrous oxide emissions from soils: How well do we understand the processes and their controls? Philosophical Transactions of the Royal Society B, 368, 20130122. 10.1098/rstb.2013.0122 PMC3682742 23713120

[gcb15884-bib-0008] Canada EaCC . (2020). National inventory report 1990–2018: Greenhouse gas sources and sinks in Canada. Canada's submission to the UNFCCC. https://unfccc.int/documents/224829

[gcb15884-bib-0009] Cayuela, M. L. , Aguilera, E. , Sanz‐Cobena, A. , Adams, D. C. , Abalos, D. , Barton, L. , Ryals, R. , Silver, W. L. , Alfaro, M. A. , Pappa, V. A. , Smith, P. , Garnier, J. , Billen, G. , Bouwman, L. , Bondeau, A. , & Lassaletta, L. (2017). Direct nitrous oxide emissions in Mediterranean climate cropping systems: Emission factors based on a meta‐analysis of available measurement data. Agriculture, Ecosystems & Environment, 238, 25–35. 10.1016/j.agee.2016.10.006

[gcb15884-bib-0010] Charles, A. , Rochette, P. , Whalen, J. K. , Angers, D. A. , Chantigny, M. H. , & Bertrand, N. (2017). Global nitrous oxide emission factors from agricultural soils after addition of organic amendments: A meta‐analysis. Agriculture, Ecosystems & Environment, 236, 88–98.

[gcb15884-bib-0011] China . (2018). The people's republic of China second biennial update report on climate change. https://unfccc.int/sites/default/files/resource/China%202BUR_English.pdf

[gcb15884-bib-0012] Davidson, E. A. , Keller, M. , Erickson, H. E. , Verchot, L. V. , & Veldkamp, E. (2000). Testing a conceptual model of soil emissions of nitrous and nitric oxides. BioScience, 50, 667–680.

[gcb15884-bib-0013] De Klein, C. , Novoa, R. S. A. , Ogle, S. , Smith, K. A. , Rochette, P. , Wirth, T. C. , McConkey, B. G. , Mosier, A. , & Rypdal, K. (2006). Chapter 11—N_2_O emissions from managed soils, and CO_2_ emissions from lime and urea application. In S. Eggleston , L. Buendia , K. Miwa , T. Ngara , & K. Tanabe (Eds.), V4 agriculture, forestry and other land use, 2006 IPCC guidelines for national greenhouse gas inventories (pp. 11.1–11.54). Institute for Global Environmental Strategies.

[gcb15884-bib-0014] Di Rienzo, J. A. , Casanoves, F. , Balzarini, M. G. , Gonzalez, L. , Tablada, M. , & Robledo, C. W. (2017). InfoStat versión 2017. InfoStat Group, Facultad de Ciencias Agropecuarias, Universidad Nacional de Córdoba. http://www.infostat.com.ar

[gcb15884-bib-0015] Eagle, A. J. , Mclellan, E. L. , Brawner, E. M. , Chantigny, M. H. , Davidson, E. A. , Dickey, J. B. , Linquist, B. A. , Maaz, T. M. , Pelster, D. E. , Pittelkow, C. M. , van Kessel, C. , Vyn, T. J. , & Cassman, K. G. (2020). Quantifying on‐farm nitrous oxide emission reductions in food supply chains. Earth's Future, 8, e2020EF001504.

[gcb15884-bib-0016] Firestone, M. K. , & Davidson, E. A. (1989). Microbiological basis of NO and N_2_O production and consumption in soils. In M. O. Andreae & D. S. Schimel (Eds.), Exchange of trace gases between terrestrial ecosystems and the atmosphere (pp. 7–21). John Wiley and Sons Ltd. https://hero.epa.gov/hero/index.cfm/reference/details/reference_id/92805

[gcb15884-bib-0017] Gałecki, A. , & Burzykowski, T. (2013). Linear mixed‐effects models using R: A step‐by‐step approach. Springer.

[gcb15884-bib-0018] Gelfand, I. , Shcherbak, I. , Millar, N. , Kravchenko, A. N. , & Robertson, G. P. (2016). Long‐term nitrous oxide fluxes in annual and perennial agricultural and unmanaged ecosystems in the upper Midwest USA. Global Change Biology, 22, 3594–3607. 27510313 10.1111/gcb.13426

[gcb15884-bib-0019] Gerber, J. S. , Carlson, K. M. , Makowski, D. Mueller, N. D. , Garcia, I. , de Cortazar‐Atauri, P. , Havlík, M. H. , Launay, M. , O'Connell, C. S. , Smith, P. , & West, P. C. (2016). Spatially explicit estimates of N_2_O emissions from croplands suggest climate mitigation opportunities from improved fertilizer management. Global Change Biology, 22, 3383–3394. 27185532 10.1111/gcb.13341

[gcb15884-bib-0020] Grace, P. , Shcherbak, I. , Macdonald, B. , Scheer, C. , & Rowlings, D. (2016). Emission factors for estimating fertiliser‐induced nitrous oxide emissions from clay soils in Australia’s irrigated cotton industry. Soil Research, 54, 598–603. 10.1071/SR16091

[gcb15884-bib-0021] Granli, T. , & Bøckman, O. C. (1996). Nitrous oxide (N_2_O) emissions from soils in warm climates. In N. Ahmad (Ed.), Nitrogen economy in tropical soils: Proceedings of the international symposium on nitrogen economy in tropical soils, held in Trinidad, W.I., January 9–14, 1994 (pp. 159–164). Springer.

[gcb15884-bib-0022] Groffman, P. M. , Butterbach‐Bahl, K. , Fulweiler, R. W. , Gold, A. J. , Morse, J. L. , Stander, E. K. , Tague, C. , Tonitto, C. , & Vidon, P. (2009). Challenges to incorporating spatially and temporally explicit phenomena (hotspots and hot moments) in denitrification models. Biogeochemistry, 93, 49–77. 10.1007/s10533-008-9277-5

[gcb15884-bib-0023] Hénault, C. , Bourennane, H. , Ayzac, A. , Ratié, C. , Saby, N. P. A. , Cohan, J.‐P. , Eglin, T. , & Gall, C. L. (2019). Management of soil pH promotes nitrous oxide reduction and thus mitigates soil emissions of this greenhouse gas. Scientific Reports, 9, 20182. 10.1038/s41598-019-56694-3 31882900 PMC6934481

[gcb15884-bib-0024] Hénault, C. , Grossel, A. , Mary, B. , Roussel, M. , & Léonard, J. (2012). Nitrous oxide emission by agricultural soils: A review of spatial and temporal variability for mitigation. Pedosphere, 22, 426–433. 10.1016/S1002-0160(12)60029-0

[gcb15884-bib-0025] Hergoualc’h, K. , Akiyama, H. , Bernoux, M. , Chirinda, N. , del Prado, A. , Kasimir, A. , MacDonald, J. D. , Ogle, S. M. , Regina, K. , & van der Weerden, T. J. (2019). Chapter 11: N_2_O emissions from managed soils and CO_2_ emissions from lime and urea application. In E. Calvo Buendia , K. Tanabe , A. Kranjc , J. Baasansuren , M. S. N. Fukuda , A. Osako , Y. Pyrozhenko , P. Shermanau , & S. Federici (Eds.), 2019 Refinement to the 2006 IPCC guidelines for national greenhouse gas inventories—Volume 4 agriculture, forestry and other land use (pp. 11.1–11.48). Intergovernmental Panel on Climate Change.

[gcb15884-bib-0026] Hergoualc'h, K. , Skiba, U. , Harmand, J.‐M. , & Oliver, R. (2007). Processes responsible for the nitrous oxide emission from a Costa Rican Andosol under a coffee agroforestry plantation. Biology and Fertility of Soils, 43, 787–795. 10.1007/s00374-007-0168-z

[gcb15884-bib-0027] Herrero, M. , Havlik, P. , Valin, H. , Notenbaert, A. , Rufino, M. C. , Thornton, P. K. , Blummel, M. , Weiss, F. , Grace, D. , & Obersteiner, M. (2013). Biomass use, production, feed efficiencies, and greenhouse gas emissions from global livestock systems. Proceedings of the National Academy of Sciences of the United States of America, 110, 20888–20893. 10.1073/pnas.1308149110 24344273 PMC3876224

[gcb15884-bib-0028] Hoben, J. P. , Gehl, R. J. , Millar, N. , Grace, P. R. , & Robertson, G. P. (2011). Nonlinear nitrous oxide (N_2_O) response to nitrogen fertilizer in on‐farm corn crops of the US Midwest. Global Change Biology, 17, 1140–1152. 10.1111/j.1365-2486.2010.02349.x

[gcb15884-bib-0029] Hox, J. J. (1998). Multilevel modeling: When and why. In I. Balderjahn , R. Mathar , & M. Schader (Eds.), Classification, data analysis, and data highways (pp. 147–154). Springer Verlag.

[gcb15884-bib-0030] Janssens‐Maenhout, G. , Crippa, M. , Guizzardi, D. , Muntean, M. , Schaaf, E. , Dentener, F. , Bergamaschi, P. , Pagliari, V. , Olivier, J. G. J. , Peters, J. A. H. W. , van Aardenne, J. A. , Monni, S. , Ulrike Doering, A. M. , Petrescu, R. , Solazzo, E. , & Oreggioni, G. D. (2019). EDGAR v4.3.2 Global atlas of the three major greenhouse gas emissions for the period 1970–2012. Earth System Science Data, 11, 959–1002.

[gcb15884-bib-0032] Jia, G. , Shevliakova, E. , Artaxo, P. , De‐Docoudré, N. , Houghton, R. , House, J. , Kitajima, K. , Lennard, C. , Popp, A. , Sirin, A. , Sukumar, R. , Verchot, L. , & Sporre, M. (2019). Land‐climate interactions. In P. R. Shukla , J. Skea , E. Calvo Buendia , V. Masson‐Delmotte , H.‐O. Pörtner , D. C. Roberts , P. Zhai , R. Slade , S. Connors , R. van Diemen , M. Ferrat , E. Haughey , S. Luz , S. Neogi , M. Pathak , J. Petzold , J. Portugal Pereira , P. Vyas , E. Huntley , K. Kissick , M. Belkacemi , & J. Malley (Eds.), Special report on climate change and land: An IPCC special report on climate change, desertification, land degradation, sustainable land management, food security, and greenhouse gas fluxes in terrestrial ecosystems (pp. 133–206). IPCC. https://www.ipcc.ch/site/assets/uploads/sites/4/2021/07/05_Chapter‐2‐V6.pdf

[gcb15884-bib-0034] Knowles, R. (1982). Denitrification. Microbiological Reviews, 46, 43–70. 7045624 10.1128/mr.46.1.43-70.1982 PMC373209

[gcb15884-bib-0035] Liu, S. , Lin, F. , Wu, S. , Ji, C. , Sun, Y. I. , Jin, Y. , Li, S. , Li, Z. , & Zou, J. (2017). A meta‐analysis of fertilizer‐induced soil NO and combined with N_2_O emissions. Global Change Biology, 23, 2520–2532. 27570182 10.1111/gcb.13485

[gcb15884-bib-0036] Ministry of Environment and Forests GOI . (2012). India. Second national communication to the United Nations framework convention on climate change. https://unfccc.int/resource/docs/natc/indnc2.pdf

[gcb15884-bib-0037] Morley, N. , & Baggs, E. M. (2010). Carbon and oxygen controls on N_2_O and N_2_ production during nitrate reduction. Soil Biology and Biochemistry, 42, 1864–1871. 10.1016/j.soilbio.2010.07.008

[gcb15884-bib-0038] Mueller, N. D. , Gerber, J. S. , Johnston, M. , Ray, D. K. , Ramankutty, N. , & Foley, J. A. (2012). Closing yield gaps through nutrient and water management. Nature, 490, 254–257. 10.1038/nature11420 22932270

[gcb15884-bib-0039] Novoa, R. , & Tejeda, H. R. (2006). Evaluation of the N_2_O emissions from N in plant residues as affected by environmental and management factors. Nutrient Cycling in Agroecosystems, 75, 29–46.

[gcb15884-bib-0040] Ogle, S. M. , Buendia, L. , Butterbach‐Bahl, K. , Breidt, F. J. , Hartman, M. , Yagi, K. , Nayamuth, R. , Spencer, S. , Wirth, T. , & Smith, P. (2013). Advancing national greenhouse gas inventories for agriculture in developing countries: Improving activity data, emission factors, and software technology. Environmental Research Letters, 8, 015030. 10.1088/1748-9326/8/1/015030

[gcb15884-bib-0041] Ogle, S. M. , Butterbach‐Bahl, K. , Cardenas, L. , Skiba, U. , & Scheer, C. (2020). From research to policy: Optimizing the design of a national monitoring system to mitigate soil nitrous oxide emissions. Current Opinion in Environmental Sustainability, 47, 28–36. 10.1016/j.cosust.2020.06.003

[gcb15884-bib-0042] Oktarita, S. , Hergoualc’h, K. , Anwar, S. , & Verchot, L. V. (2017). Substantial N_2_O emissions from peat decomposition and N fertilization in an oil palm plantation exacerbated by hotspots. Environmental Research Letters, 12, 104007.

[gcb15884-bib-0043] Phalan, B. , Bertzky, M. , Butchart, S. H. M. , Donald, P. F. , Scharlemann, J. P. W. , Stattersfield, A. J. , & Balmford, A. (2013). Crop expansion and conservation priorities in tropical countries. PLoS ONE, 8, e51759. 10.1371/journal.pone.0051759 23326316 PMC3541398

[gcb15884-bib-0044] Philibert, A. , Loyce, C. , & Makowski, D. (2012). Quantifying uncertainties in N_2_O emission due to N fertilizer application in cultivated areas. PLoS ONE, 7, 1–9. 10.1371/journal.pone.0050950 PMC3511396 23226430

[gcb15884-bib-0045] Portmann, F. T. , Siebert, S. , & Döll, P. (2010). MIRCA2000—Global monthly irrigated and rainfed crop areas around the year 2000: A new high‐resolution data set for agricultural and hydrological modeling. Global Biogeochemical Cycles, 24. 10.1029/2008GB003435

[gcb15884-bib-0046] R Core Team . (2020). R: A language and environment for statistical computing. R Foundation for Statistical Computing. https://www.R‐project.org/

[gcb15884-bib-0047] Reddy, S. , Panichelli, L. , Waterworth, R. M. , Federici, S. , Green, C. , Jonckheere, I. , Kahuri, S. , Kurz, W. A. , de Ligt, R. , Ometto, J. P. , Petersson, H. , Takahiro, E. , Paul, T. , Tullis, J. , Somogyi, Z. , Pandya, M. , Rocha, M. T. , & Suzuki, K. (2019). Chapter 3: Consistent representation of land. In E. Calvo Buendia , K. Tanabe , A. Kranjc , J. Baasansuren , M. Fukuda , S. Ngarize , A. Osako , Y. Pyrozhenko , P. Shermanau , & S. Federici (Eds.), 2019 Refinement to the 2006 IPCC guidelines for national greenhouse gas inventories—Volume 4 agriculture, forestry and other land use (pp. 3.1–3.55). Intergovernmental Panel on Climate Change.

[gcb15884-bib-0048] Rochette, P. , Liang, C. , Pelster, D. , Bergeron, O. , Lemke, R. , Kroebel, R. , MacDonald, D. , Yan, W. , & Flemming, C. (2018). Soil nitrous oxide emissions from agricultural soils in Canada: Exploring relationships with soil, crop and climatic variables. Agriculture, Ecosystems and Environment, 254, 69–81. 10.1016/j.agee.2017.10.021

[gcb15884-bib-0049] Shcherbak, I. , Millar, N. , & Robertson, G. P. (2014). A global meta‐analysis of the nonlinear response of soil nitrous oxide (N_2_O) emissions to fertilizer nitrogen. Proceedings of the National Academy of Sciences of the United States of America, 111, 9199–9204. 10.1073/pnas.1322434111 24927583 PMC4078848

[gcb15884-bib-0050] Skiba, U. , Hergoualc’h, K. , Drewer, J. , Meijide, A. , & Knohl, A. (2020). Oil palm plantations are large sources of nitrous oxide, but where are the data to quantify the impact on global warming? Current Opinion in Environmental Sustainability, 47, 81–88. 10.1016/j.cosust.2020.08.019

[gcb15884-bib-0051] Skiba, U. , & Smith, K. A. (2000). The control of nitrous oxide emissions from agricultural and natural soils. Chemosphere: Global Change Science, 2, 379–386.

[gcb15884-bib-0052] Stehfest, E. , & Bouwman, L. (2006). N_2_O and NO emission from agricultural fields and soils under natural vegetation: Summarizing available measurement data and modeling of global annual emissions. Nutrient Cycling in Agroecosystems, 74, 207–228. 10.1007/s10705-006-9000-7

[gcb15884-bib-0053] Thompson, K. A. , Bent, E. , Abalos, D. , Wagner‐Riddle, C. , & Dunfield, K. E. (2016). Soil microbial communities as potential regulators of in situ N_2_O fluxes in annual and perennial cropping systems. Soil Biology and Biochemistry, 103, 262–273. 10.1016/j.soilbio.2016.08.030

[gcb15884-bib-0054] Tian, H. , Xu, R. , Canadell, J. G. , Thompson, R. L. , Winiwarter, W. , Suntharalingam, P. , Davidson, E. A. , Ciais, P. , Jackson, R. B. , Janssens‐Maenhout, G. , Prather, M. J. , Regnier, P. , Pan, N. , Pan, S. , Peters, G. P. , Shi, H. , Tubiello, F. N. , Zaehle, S. , Zhou, F. , … Yao, Y. (2020). A comprehensive quantification of global nitrous oxide sources and sinks. Nature, 586, 248–256. 10.1038/s41586-020-2780-0 33028999

[gcb15884-bib-0055] Tubiello, F. N. , Salvatore, M. , Rossi, S. , Ferrara, A. , Fitton, N. , & Smith, P. (2013). The FAOSTAT database of greenhouse gas emissions from agriculture. Environmental Research Letters, 8, 015009.

[gcb15884-bib-0056] USDA . (2017). Chapter 3. Examination and description of soil profiles. In C. Ditzler , K. Scheffe , & H. C. Monger (Eds.), Soil science division staff. Soil survey manual. USDA Handbook (18 pp). Government Printing Office.

[gcb15884-bib-0057] US‐EPA . (2021). Inventory of U.S. greenhouse gas emissions and. Environmental Protection Agency https://www.epa.gov/ghgemissions/inventory‐us‐greenhouse‐gas‐emissions‐and‐sinks‐1990‐2019

[gcb15884-bib-0058] van der Weerden, T. J. , Cox, N. , Luo, J. , Di, H. J. , Podolyan, A. , Phillips, R. L. , Saggar, S. , de Klein, C. , Ettema, P. , & Rys, G. (2016). Refining the New Zealand nitrous oxide emission factor for urea fertiliser and farm dairy effluent. Agriculture, Ecosystems & Environment, 222, 133–137. 10.1016/j.agee.2016.02.007

[gcb15884-bib-0059] van Groenigen, J. W. , Velthof, G. L. , Oenema, O. , Van Groenigen, K. J. , & Van Kessel, C. (2010). Towards an agronomic assessment of N_2_O emissions: A case study for arable crops. European Journal of Soil Science, 61, 903–913. 10.1111/j.1365-2389.2009.01217.x

[gcb15884-bib-0060] van Lent, J. , Hergoualc’h, K. , & Verchot, L. V. (2015). Soil N_2_O and NO emissions from land use and land‐use change in the tropics and subtropics: A meta‐analysis. Biogeosciences, 12, 7299–7313.

[gcb15884-bib-0061] Vico, G. , & Brunsell, N. A. (2018). Tradeoffs between water requirements and yield stability in annual vs. perennial crops. Advances in Water Resources, 112, 189–202. 10.1016/j.advwatres.2017.12.014

[gcb15884-bib-0062] Wang, Y. , Guo, J. , Vogt, R. D. , Mulder, J. , Wang, J. , & Zhang, X. (2018). Soil pH as the chief modifier for regional nitrous oxide emissions: New evidence and implications for global estimates and mitigation. Global Change Biology, 24, e617–e626. 10.1111/gcb.13966 29171128

[gcb15884-bib-0063] Wells, K. C. , Millet, D. B. , Bousserez, N. , Henze, D. K. , Griffis, T. J. , Chaliyakunnel, S. , Dlugokencky, E. J. , Saikawa, E. , Xiang, G. , Prinn, R. G. , O'Doherty, S. , Young, D. , Weiss, R. F. , Dutton, G. S. , Elkins, J. W. , Krummel, P. B. , Langenfelds, R. , & Paul Steele, L. (2018). Top‐down constraints on global N_2_O emissions at optimal resolution: Application of a new dimension reduction technique. Atmospheric Chemistry and Physics, 18, 735–756.

[gcb15884-bib-0064] West, P. C. , Gerber, J. S. , Engstrom, P. M. , Mueller, N. D. , Brauman, K. A. , Carlson, K. M. , Cassidy, E. S. , Johnston, M. , MacDonald, G. K. , Ray, D. K. , & Siebert, S. (2014). Leverage points for improving global food security and the environment. Science, 345, 325–328. 10.1126/science.1246067 25035492

[gcb15884-bib-0065] Zhang, X. , Fang, Q. , Zhang, T. , Ma, W. , Velthof, G. L. , Hou, Y. , Oenema, O. , & Zhang, F. (2020). Benefits and trade‐offs of replacing synthetic fertilizers by animal manures in crop production in China: A meta‐analysis. Global Change Biology, 26, 888–900. 10.1111/gcb.14826 31495039

[gcb15884-bib-0066] Zhang, X. , Zou, T. , Lassaletta, L. , Mueller, N. D. , Tubiello, F. N. , Lisk, M. D. , Lu, C. , Conant, R. T. , Dorich, C. D. , Gerber, J. , Tian, H. , Bruulsema, T. , Maaz, T. M. C. , Nishina, K. , Bodirsky, B. L. , Popp, A. , Bouwman, L. , Beusen, A. , Chang, J. , … Davidson, E. A. (2021). Quantification of global and national nitrogen budgets for crop production. Nature Food, 2(7), 529–540. 10.1038/s43016-021-00318-5 37117677

[gcb15884-bib-0067] Zhou, M. , Zhu, B. , Wang, S. , Zhu, X. , Vereecken, H. , & Brüggemann, N. (2017). Stimulation of N_2_O emission by manure application to agricultural soils may largely offset carbon benefits: A global meta‐analysis. Global Change Biology, 23, 4068–4083. 28142211 10.1111/gcb.13648

[gcb15884-bib-0068] Zhu, Y. , Merbold, L. , Leitner, S. , Xia, L. , Pelster, D. E. , Diaz‐Pines, E. , Abwanda, S. , Mutuo, P. M. , & Butterbach‐Bahl, K. (2020). Influence of soil properties on N_2_O and CO_2_ emissions from excreta deposited on tropical pastures in Kenya. Soil Biology and Biochemistry, 140, 107636. 10.1016/j.soilbio.2019.107636

