# Supplementary material for: Improved accuracy and reduced uncertainty in greenhouse gas inventories by refining the IPCC emission factor for direct N2O emissions from nitrogen inputs to managed soils
Source: Glob Chang Biol. 2021 Sep 25;27(24):6536–50. doi: 10.1111/gcb.15884 (PMC9293294; doi:10.1111/gcb.15884)
Supplement: Supplementary file 1 — Supplementary Material [file GCB-27-6536-s001.pdf]

Supplementary information for the paper

Improved accuracy and reduced uncertainty in greenhouse gas inventories by refining the IPCC emission factor for direct N_2O emissions from nitrogen inputs to managed soils

Kristell Hergoualc'h[1*], Nathan Mueller[2,3], Martial Bernoux[4], Äsa Kasimir[5], Tony J. van der Weerden[6], Stephen M. Ogle[2,7]

[1] Center for International Forestry Research (CIFOR), Lima, Peru
[2] Department of Ecosystem Science and Sustainability, Colorado State University, Fort Collins, CO, USA
[3] Department of Soil and Crop Sciences, Colorado State University, Fort Collins, CO, USA
[4] Food and Agriculture Organization of the United Nations (FAO), Rome, Italy
[5] University of Gothenburg
[6] AgResearch Ltd, Invermay Agricultural Centre, Mosgiel, New Zealand
[7] Natural Resource Ecology Laboratory, Colorado State University, Fort Collins, CO, USA
* Corresponding author. Kristell Hergoualc'h, Center for International Forestry Research (CIFOR) c/o Centro Internacional de la Papa (CIP), Av. La Molina 1895, La Molina, Apdo Postal 1558, 15024 Lima, Peru. Email: k.hergoualch@cgiar.org

TABLE S1 Sample size, mean, and uncertainty range of the EF_1 as influenced by climate, fertilizer form, land cover, and topsoil texture class, and C content. A and B indicate a significant difference between means for a given factor based on LSD Fisher test

Factor	Class	n	Mean	95% C.I.
**Climate**	Temperate/boreal wet	524	0.014 ^{B}	0.008 – 0.020
	Temperate/boreal dry	121	0.007 ^{AB}	-0.002 – 0.015
	Tropical wet	117	0.015 ^{AB}	0.011 – 0.018
	Tropical dry	86	0.004 ^{A}	-0.004 – 0.013
**Fertilizer form**	Synthetic	602	0.014 ^{B}	0.007 – 0.020
	Mixed synthetic and organic	48	0.014 ^{AB}	0.011 – 0.017
	Organic	162	0.007 ^{A}	0.003 – 0.012
**Land cover**	Annual croplands	543	0.014 ^{B}	0.011 – 0.018
	Bare soils	74	0.012 ^{AB}	0.004 – 0.019
	Perennial systems	231	0.009 ^{A}	0.005 – 0.013
**Texture class**	Fine	131	0.023 ^{B}	0.018 – 0.028
	Medium	571	0.010 ^{A}	0.007 – 0.013
	Coarse	30	0.006 ^{A}	-0.005 – 0.016
**Soil C content**	High (≥ 2%)	265	0.015 ^{B}	0.012 – 0.019
	Medium (1-2%)	241	0.007 ^{A}	0.003 – 0.010
	Low (< 1%)	159	0.009 ^{A}	0.004 – 0.013

TABLE S2 Global agricultural fertilizer N consumption, and also countries with the largest inputs of fertilizer N to croplands (synthetic – manure). The dataset combines synthetic N application rates by Mueller *et al.* (2012) and manure N application rates by West *et al.* (2014) from circa 2000

	Fertilizer N application (Gg N)		
	**Total Fertilizer**	**Synthetic Fertilizer**	**Manure Fertilizer**
**Global Agriculture**	103,499.3	69,624.3	33,875.0
**China**	26,183.5	19,927.8	6,255.7
**India**	15,059.2	9,312.9	5,746.3
**United States**	13,515.6	9,521.7	3,993.9
**Brazil**	2,948.0	1,517.7	1,430.3
**Pakistan**	2,754.2	2,067.4	686.8
**Indonesia**	2,331.3	1,647.4	683.9
**France**	2,206.7	1,707.5	499.2
**Mexico**	2,087.3	1,167.5	919.8
**Canada**	1,971.8	1,564.2	407.6
**Germany**	1,865.1	1,372.4	492.7

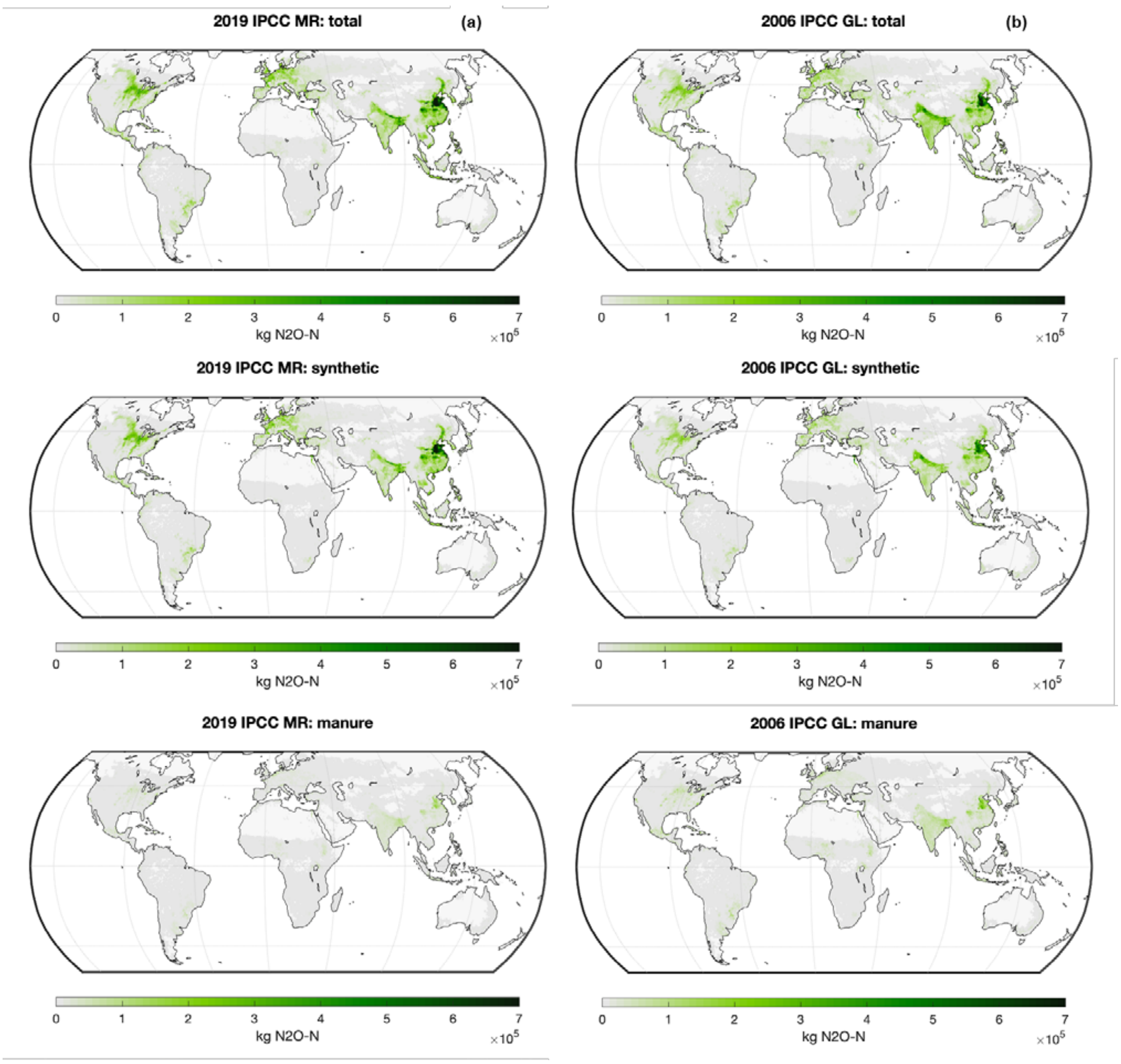

FIGURE S1 Direct soil N_2O emissions from global agricultural croplands using the Tier 1 method from the 2019 IPCC Methods Refinement to the 2006 IPCC National GHG Inventories Guidelines (2019 IPCC MR) (a) and the 2006 IPCC National GHG Inventories Guidelines (2006 IPCC GL) (b). The top Figures display emissions from both synthetic and manure application (total), the middle and bottom Figures refer to synthetic and manure application separately
